# Supplementary material for: Community perception of school-based mass drug administration program for soil-transmitted helminths and Schistosomiasis in Ogun State, Nigeria
Source: PLoS Negl Trop Dis. 2023 Jul 17;17(7):e0011213. doi: 10.1371/journal.pntd.0011213 (PMC10374069; doi:10.1371/journal.pntd.0011213)
Supplement: S1 File — (PDF) [file pntd.0011213.s001.pdf]

COSTRONS STUDY KEY INFORMANT INTERVIEW GUIDE

Interview Guide

(Expected participants: Community leaders and Community NTD Focal person)

<u>Introduction</u>

(Please remind the client about the audio recording and not to use real names when discussing their perception and thoughts). Thank you for talking with me today. I am interested in learning more about your thoughts and perception as a …………………. on the of……………Mass Drug Administration (MDA) programs in Nigeria. I am going to ask you some questions about these topics. Please know that there is no right or wrong answer to any of the questions that I will ask. Beyond asking the questions, the discussion will be entirely driven by your responses – my only goal is to facilitate to get more clarity on the statements you share.

During our conversation, you will notice that I will not give you feedback on your responses because I do not want to influence your answers. You may also notice that I will write things down on paper while you talk – this notetaking is simply to remind me to ask you a follow-up question as needed. You are under no obligation to talk about anything that you are not comfortable discussing with me.

This session is being recorded, as noted in the consent form you signed before so that none of your informative comments and feedback will be missed. Again, your names will not be collected, and your comments will be confidential. Please let me know if you have any questions or concerns before we begin.

Study ID……………………………………..

COSTRONS STUDY KEY INFORMANT INTERVIEW GUIDE

Section A: Basic Information of the Respondent

(Either have the participant complete on paper themselves or the Interviewer will ask these questions and fill out the form accordingly)

1. Age at last birthday…………………………………………………………………………………….
2. Gender: Female ( )   Male ( )
3. Religion………………………………………………………………………………………………..
4. Ethnic group (1) Hausa (2) Igbo (3) Yoruba (4) Other …………………….…………………………...
5. Education: No formal school/ Primary/ Secondary/ Tertiary /Others………………………………………
6. Marital Status: Single ( ) Married( ) Separated (   ) Divorced(   ) Widowed( )
7. Occupation…………………………………………………………………………………………..
8. Monthly Income……………………………………………………………………………………..

Section B: Knowledge of Neglected Tropical Diseases (NTDs)

9. What is the scope of your work?
   Probe: a) Describe your role and what the responsibilities attached to it
   b) Describe what you do if you are not directly involved.
10. Could you list the current top five priorities of the health sector in your district?
11. In your opinion, what is the level of importance of treating these neglected tropical diseases for your district?
    Probe: a) High priority/Medium priority/Low priority/Don't know
12. How frequent is the occurrence of STH and Schistosomiasis in your community in your own words?
    Guide: use local names of diseases for easy understanding
13. How conversant are you with school-based MDA control program in your community that is meant to mitigate these diseases (refer to the diseases earlier mentioned)?
    Probe: a) Know very much/Know a little/Know nothing
14. How well are the community stakeholders cooperative in the execution of the school-based MDA control programs?
    Probe: a) Very cooperative/Barely cooperative/Not cooperative at all
15. What more could be done to make the integration or cooperation of community stakeholders flawless for the achievement of the NTD goals?

COSTRONS STUDY KEY INFORMANT INTERVIEW GUIDE

Section C: Communication, Dissemination and Data Management

16. How well is communication effective between stakeholders at the community level and the state team?

    Probe: a) Very effective/ Barely effective/ Not effective at all
17. What barriers do you think could prevent school based MDA programs in your community?
18. What factors do you think will help school based MDA programs in your community?
19. How is information communicated between the stakeholders and sponsors of the program and other stakeholders at the state and federal level?

    Probe: a) How easy, how effective, what method is used to send/ communicate data

    b) State level

    c) Federal level
20. How well do recipients of the program embrace the school based MDA programs?

    Probe: a) Recipients usually are - hesitant/are eager/are indifferent
21. Are there rumours that exist about the drugs being administered to students or school-based MDA programs?

    Probe: a) please mention some
22. If yes, how are they being handled in the community?
23. How well is data documented on the school-based MDA programs at the community level?

    Probe: a) Is there a register available to keep records
24. Are they referred to the state level for treatment
25. How often are data reconciled to the state ministry of health and other stakeholders on the school-based MDA programs?

    Probe: a) Weekly/Monthly/Quarterly/Yearly/When we have any new case/Whenever they come for data reconciliation
26. Are there legislations or policies you are aware of that hinders the improvement of school-based MDA programs?
27. Do you have suggestions on what you think might help facilitate the improvement of the school-based MDA programs?

Thank you for your time and sharing your thoughts with me.
