# Supplementary material for: Community perception of school-based mass drug administration program for soil-transmitted helminths and Schistosomiasis in Ogun State, Nigeria
Source: PLoS Negl Trop Dis. 2023 Jul 17;17(7):e0011213. doi: 10.1371/journal.pntd.0011213 (PMC10374069; doi:10.1371/journal.pntd.0011213)
Supplement: S2 File — (PDF) [file pntd.0011213.s002.pdf]

COSTRONS STUDY
FOCUS GROUP DISCUSSION GUIDE

Focus Group Discussion (FGD) Guide

(Expected participants: Teachers, Parents, Town announcers, and Opinion Leaders)

Introduction

(Please remind the client about the audio recording and not to use real names when discussing their perception and thoughts). Thank you for talking with me today. I am interested in learning more about your thoughts and perception as a teacher/parent on the facilitators and barriers to the uptake and accessing Mass Drug Administration (MDA) programs in Nigeria. I am going to ask you some questions about these topics. Please know that there is no right or wrong answer to any of the questions that I will ask. Beyond asking the questions, the discussion will be entirely driven by your responses – my only goal is to facilitate getting more clarity on the statements you share.

During our conversation, you will notice that I will not give you feedback on your responses because I do not want to influence your answers. You may also notice that I will write things down on paper while you talk – this note-taking is simply to remind me to ask you a follow-up question as needed. You are under no obligation to talk about anything that you are not comfortable discussing with me.

This session is being recorded, as noted in the consent form you signed before so that none of your informative comments and feedback will be missed. Again, your names will not be collected, and your comments will be confidential. Please let me know if you have any questions or concerns before we begin.

Study ID……………………………………..

COSTRONS STUDY
FOCUS GROUP DISCUSSION GUIDE

Section A: Basic Information of the Respondent

(Either have the participant complete on paper themselves or the Interviewer will ask these questions and fill out the form accordingly)

1. Age at last birthday……………………………………………………………………………………….
2. Gender: Female ( ) Male ( )
3. Religion…………………………………………………………………………………………………..
4. Ethnic group (1) Hausa (2) Igbo (3) Yoruba (4) Other ………………………………………………...
5. Education: No formal school/ Primary/ Secondary/ Tertiary /Others………………………………………
6. Marital Status: Single ( ) Married( ) Separated ( ) Divorced( ) Widowed( )
7. Occupation……………………………………………………………………………………………..
8. Monthly
9. Income…………………………………………………………………………………………..

Section B: Knowledge of Schistosomiasis and Soil Helminthes (STH)

10. What are the common illnesses in your community?
11. Are these illnesses common in your community?

    Probe: Ask specifically about for schistosomiasis, soil transmitted Helminthes (STH)
12. Could you please describe what you know about?

    Probe: a) Schistosomiasis
13. What are the local names of STH and schistosomiasis?
14. What are the causes
15. What are the signs/symptoms of this disease?
16. Could you please describe what you know about Soil Helminthes (STH)?

    Probe: a) What is the local name for STH and schistosomiasis
17. What are the causes
18. What are the signs/symptoms of the disease
19. How serious can these infections be?

    Probe: a) Ask about their knowledge of the above question

    b) Is there treatment?

    c) Is there a cure?
20. How soon can they have access to treatment/ a cure
21. Do you know people who are infected with Schistosomiasis in this community?

    Probe: a) How did you identify that it was Schistosomiasis they were infected with
22. In the last year, how many people do you think have had Schistosomiasis

23. In this community, what category of people are more infected with Schistosomiasis
24. Why do you think this category/these categories of people were infected?
25. Do you know people who are infected with Soil Helminthes in this community?

    Probe: a) How did you identify that it was Soil Helminthes they were infected with

    b) In the last year, how many people do you think have had Soil Helminthes?

    c) In this community, what category of people are more infected with Soil Helminthes

    d) Why do you think this category/these categories of people were infected?

26. How do you think people in the community contract Schistosomiasis and Soil Helminthes (STH)?

    Probe for effectiveness and preference among such things mentioned.
27. How do people treat those infected with this/these disease(s)? -Is this in terms of health treatment of psychosocial disposition?
28. What are the best treatment options available for these diseases?

    Probe: a) Please mention the options

    b) How much does each option cost?

    c) In your community, what strategy is adopted for treatment of these diseases?

<u>Section C: Treatment and Access to Care</u>

29. What effort has the community made to report the problem of schistosomiasis and STH to the Local Government health authorities for necessary assistance?

    Probe: a) Ask for any past or recent efforts.
30. Are drugs available in your community for treating Neglected Tropical Diseases (NTDs)?

    Probe: a) Please describe the drugs/state their names if you know? (Guide-praziquantel, ivermectin, albendazole etc.)

    b) How much do they cost

    c) Are people willing to pay, please give reasons for this answer

    d) How many times is treatment/drugs provided and/or supplied in a year
31. Are you aware of programs through which drugs are administered to address these diseases in your community? If yes, please explain the program(s).

    Probe: a) who do you think is responsible for the program/access?
32. Please kindly describe how the community was involved in the program(s).

    Probe:a) what are the roles/contributions of individuals/volunteers, households and community?

    b) How do they get involved?

    c) Are they paid to render this service/help

33. What is your perception of the success of past and ongoing program(s) on NTD control earlier mentioned/discussed?

    Probe: a) What ideas do you think can help in implementing this program in your community?

    b) What ideas do you think will prevent this program in your community?

    c) How can such programs be sustained by the community?

Thank you for your time and sharing your thoughts with us.
