# Supplementary material for: Community perception of school-based mass drug administration program for soil-transmitted helminths and Schistosomiasis in Ogun State, Nigeria
Source: PLoS Negl Trop Dis. 2023 Jul 17;17(7):e0011213. doi: 10.1371/journal.pntd.0011213 (PMC10374069; doi:10.1371/journal.pntd.0011213)
Supplement: S3 File — (PDF) [file pntd.0011213.s003.pdf]

COSTRONS STUDY
FOCUS GROUP DISCUSSION GUIDE

Interview Guide

(Expected participants: School-aged children)

Introduction

(Please remind the client about the audio recording and not to use real names when discussing their perception and thoughts). Thank you for talking with me today. I am interested in learning more about what you think as a student about the uptake of Mass Drug Administration (MDA) programs in Nigeria and taking your drugs. I am going to ask you some questions about these topics. Please know that there is no right or wrong answer to any of the questions that I will ask. Beyond asking the questions, the discussion will be entirely driven by your responses – my only goal is to facilitate getting more clarity on the statements you share.

During our conversation, you will notice that I will not give you feedback on your responses because I do not want to influence your answers. You may also notice that I will write things down on paper while you talk – this note-taking is simply to remind me to ask you a follow-up question as needed. You are under no obligation to talk about anything that you are not comfortable discussing with me.

This session is being recorded, as noted in the consent form you signed before so that none of your informative comments and feedback will be missed. Again, your names will not be collected, and your comments will be confidential. Please let me know if you have any questions or concerns before we begin.

Study ID……………………………………..

COSTRONS STUDY
FOCUS GROUP DISCUSSION GUIDE

Section A: Basic Information of the Respondent

(Either have the participant complete on paper themselves or the Interviewer will ask these questions and fill out the form accordingly)

1. Age at last birthday……………………………………………………………………………………….
2. Gender: Female ( ) Male ( )
3. Religion……………………………………………………………………………………………..
4. Ethnic group (1) Hausa (2) Igbo (3) Yoruba (4) Other ……………………………………………………
5. Class:……………………………………………………………………………………………..

Section B: General Knowledge of Schistosomiasis and Soil Helminthes (STH)

5. Please tell me what you know about the worm disease?

   Probe: a) Use examples/illustrations they will understand

   b) Use local language

   c) Demonstrate where necessary
6. How can someone get it?
7. Can it be treated?
8. Is there a cure for it?
9. How do you think it can be treated?

   Probe: a) use scenarios/examples to explain
10. Where do you think someone who has the disease can get it?
11. Would you take medicine/drugs to prevent these diseases if they give you?
12. Why will you take the drugs?
13. Why will you not take the drugs?
14. What are the things that will make you not to take the drugs?
15. What are the things that will make you to take the drugs?
16. What ideas do believe will help Children take drugs for deworming?
17. What strategies do you think MDA programs should use to get children to participate in these programs?
18. Do you have anything else to tell me/us?

Thank you for your time and sharing your thoughts with us.
