# Supplementary material for: Community perception of school-based mass drug administration program for soil-transmitted helminths and Schistosomiasis in Ogun State, Nigeria
Source: PLoS Negl Trop Dis. 2023 Jul 17;17(7):e0011213. doi: 10.1371/journal.pntd.0011213 (PMC10374069; doi:10.1371/journal.pntd.0011213)
Supplement: Attachment — Submitted filename: Reviewer 2 Comments_Response.pdf [file pntd.0011213.s006.pdf]

Response to Reviewer 2

Comments

1. Is this study for the entire country or Ogun State, Nigeria? the title should indicate the specific location in Nigeria where the study was conducted.
2. the authors should explain what they meant by poor drug acceptability, accessibility and effectiveness. What instrument did they use to test the effectiveness of the drug?
3. Is it actually lack of support or inadequacy of the available support? More clarifications on this statement
4. Point of correction, schistosomiasis and STH are for elimination and not eradication.
5. Issues around drug availability, timeliness in drug logistics and distribution should have been investigated too.
6. Correct this statement, in should be replaced instead use With and affirm should be affirmed.
7. The reason for the children being able to recall the color of the drugs given to them may be attributed to their being the primary beneficiaries of this school-based MDA.

Dear Reviewer,

We would like to thank you for your valuable comments and suggestions. Below are our responses to each of the points raised:

1. Is this study for the entire country or Ogun State, Nigeria? the title should indicate the specific location in Nigeria where the study was conducted.
   The study was conducted specifically in Ogun State, Nigeria. We apologize for the oversight in not including this information in the title. We revised the title to reflect the location: "Community Perception of School-Based Mass Drug Administration Program for Soil-Transmitted Helminths and Schistosomiasis in Ogun State, Nigeria."

2. The authors should explain what they meant by poor drug acceptability, accessibility and effectiveness. What instrument did they use to test the effectiveness of the drug?
   We appreciate the reviewer's comment regarding the need to clarify the terms "poor drug acceptability, accessibility, and effectiveness." The revised manuscript reads, A semi-structured questionnaire guided the exploration of ideas, and the data were analyzed using the QRS Nvivo 12 software package. The study found several barriers, such as the influence of parents, lack of sufficient knowledge, and side effects. The study recommended strategies such as improving community sensitization and engagement, drug distribution and performance, and enhancing partner collaboration and coordination to improve the school-based mass drug administration programs.

3. Is it actually lack of support or inadequacy of the available support? More clarifications on this statement
   We acknowledge the reviewer's comment regarding the lack of clarity between "lack of support" and "inadequacy of the available support." The revised manuscript reads, “Additionally, the study found that the implementation of Neglected Tropical Disease control programs was inconsistent due to the inadequacy of available support from partners. Overall, our study provides important insights into the barriers and facilitators.”

4. Point of correction, schistosomiasis and STH are for elimination and not eradication.
   Thank you for pointing out the correction regarding the terminology. We have rectified the statement to accurately reflect that the goal for schistosomiasis and STH is elimination, not eradication. The revised

manuscript reads, “Despite significant progress in eliminating SCH and STH, there is a need to investigate the implementation challenges that hinder the uptake, uptake, and coverage of the integrated MDA programs at the community level. Understanding the community's perceptions can provide valuable insights for program managers to improve current control strategies.”

5. Issues around drug availability, timeliness in drug logistics and distribution should have been investigated too.
   We appreciate the reviewer's suggestion to investigate issues related to drug availability, timeliness in drug logistics, and distribution. We will consider this in our future research which will be focused on Health workers perspective which will include an expanded discussion on these topics, considering the potential impact on the success of the school-based MDA program.

6. Correct this statement, in should be replaced instead use With and affirm should be affirmed.
   Thank you for highlighting the error in the statement. We have made the necessary correction, the revised manuscript reads, “Regarding the treatment of SCH and STH, most respondents were aware of the importance of the treatment and affirmed that the government provides drugs for them.”

7. The reason for the children being able to recall the color of the drugs given to them may be attributed to their being the primary beneficiaries of this school-based MDA.
   The reviewer's comment regarding the reason for children being able to recall the color of the drugs given to them as primary beneficiaries is insightful. In our revised manuscript, we have, “The N/ESAC recalled the color of the drugs in contrast to the adult respondents, who could state the difference in the drugs administered for STH and SCH. This could be because the N/ESAC are the primary beneficiaries of the school-based MDA control program.”

Once again, we sincerely appreciate the thoughtful comments and suggestions provided by the reviewer, which will significantly contribute to the improvement of our manuscript.
