# Supplementary material for: Community perception of school-based mass drug administration program for soil-transmitted helminths and Schistosomiasis in Ogun State, Nigeria
Source: PLoS Negl Trop Dis. 2023 Jul 17;17(7):e0011213. doi: 10.1371/journal.pntd.0011213 (PMC10374069; doi:10.1371/journal.pntd.0011213)
Supplement: Attachment — Submitted filename: Plos review comments.docx [file pntd.0011213.s004.docx]

Community Assessment of School-Based Mass Drug Administration Program for Soil-Transmitted Helminths and Schistosomiasis in Nigeria

The study according to the abstract sought to assess the community’s perceptions of MDA for STHs and Schistosomiasis so as to identify barriers to utilization and coverage in Nigeria. Several barriers :-poor drug acceptability, accessibility, and effectiveness, low knowledge and awareness of the diseases and control interventions, inadequate community engagement and involvement, and weak health system and partner support were identified. The identified strategies for addressing the barriers included community sensitization and engagement, improving drug distribution and effectiveness, strengthening health system support, and enhancing partner collaboration and coordination.

Comments

The title is somehow misleading as it does not talk of barriers of utilization and coverage but seems to speak of assessment. It should be reworded to highlight the aim and the word perception should feature since “community assessment’ alone does not give a clear idea of what is being assessed.

The abstract needs to be re-organized to clearly give background, methods, results and conclusions sections without mixing up the contents of each section. As it is currently, the methods section is mixed up with some findings/results (lines 33-40) and the conclusion section has results in it, lines 42-43.

Utilization can be substituted with uptake. The use of the word effectiveness seems not correct, perhaps the authors mean performance.

From the findings of the study, several strategies were recommended for addressing the identified barriers rather than recommendations being identified, line 37.

Introduction section:

Sentence on lines 68-69 and 69-71 need to be restructured to avoid ending one sentence with “Nigeria” and beginning the following sentence with “Nigeria” and not to use the word “burden” twice in one sentence.

1^st^ paragraph lines 66-73 has too much repetition of STH, SCH and NTDs and the authors should reorganize it to improve its flow. Sentence on line 101-103 should also be re-organized to avoid using “infections” twice.

The sentence on line 105-107 again talks about exploring the community’s perceptions of the school-based MDA programme but there is no mention of identification of the barriers of uptake (utilization) and coverage.

Study setting section:

Provide the prevalence for STH and SCH in Ogun State as well as in the purposively selected 4 LGAs (Ikenne, Abeokuta North, Abeokuta South, and Obafemi Owode) where the study was conducted.

In the methods section please indicate the number of FGDs, KIIs and SSIs that were conducted with the various categories of study participants.

Results section

The authors have presented the 4 key thematic areas as well as the sub-themes. It is not possible to assess the effectiveness of the drug through a social science (qualitative) study. This can only be done through parasitology by collecting stool samples before and after MDA to assess the number of eggs in the stool. This needs to be thought out clearly, the authors should not put perceptions as a sub-theme of knowledge, as knowledge and perceptions are 2 different things. Maybe have a theme on knowledge of the diseases’ aetiology, and MDA then another theme on perceptions of the disease and of MDA to include drugs acceptability and accessibility, then another theme on barriers and a fourth theme on identified strategies for improved uptake and coverage (programme performance).

Lines 207- 242, the results needs to be presented in a clear way to show responses given for the questions posed guided by the themes. It appears that the question on knowledge of STH and SCH and mode of transmission were posed as just one question. Yet STH and SCH are transmitted differently and thus the 2 should have been posed separately. Kindly address this so that the responses given “urinating in the river” are not put as poor as they are partly correct.

Thus, sub themes 1.1 to 1.3 need to be re-done.

Sub theme 1.4 is put as Mass Drug Administration but it is important to indicate what components of the MDA are being investigated and reported about, is it the sensitization/ awareness creation about the campaign? Or the drugs administration? Or the drugs given and access to these drugs. Or the side effects experienced by the school-age children after consuming the drugs? Please reorganize and report each theme with the sub-themes conclusively.

Sub-theme 2.3 Drug effectiveness is misleading as what is reported is effectiveness of the distribution. Line 418, what is a big effectiveness? When the respondents give such responses, it is the responsibility of the authors to make some minor grammatical changes to allow for readability and understanding. Please check most of the quotes to ensure that they are readable and understandable.

All themes should be rethought so that each is dealt with conclusively. As presented there is too much “cross-over” of themes and the sub-themes therein.

Discussion section:

Lines 546-549 should be in the results section- avoid presenting the results in this section but discuss the results based on your study findings and those of other publications.

Lines 556-557- Our findings revealed that most participants associated these diseases with drinking dirty contaminated water (17), is poorly presented as the authors are discussing their finding and yet they are giving a reference (17, a study by Musuva *et al* that was conducted in Kenya) instead of stating what their finding in comparison with the other report (17) shows.

Restructure or allow some flow of the discussions as per the themes and sub-themes.

On study limitations, generalizability is not intended in qualitative studies. The idea is to get views, thoughts, perceptions, attitudes etc from key community members and the sample is not determined statistically. Thus, the aim of qualitative studies is to help build theory that can inform the design of a quantitative study or be able to give key information that can be used to improve performance of programmes such as MDAs. The 3^rd^ limitation should clearly state the time that had elapsed between the MDA and data collection so that recall bias as a limitation can be justifiable. I don’t think that language barrier should be a limitation as it is assumed that the data collection tools were translated and back translated to the local language and back to English by experts and that they were reviewed with the field assistants. Language barrier should not be a limitation in my opinion.
